# Status of soil-transmitted helminth infections in schoolchildren in Laguna Province, the Philippines: Determined by parasitological and molecular diagnostic techniques

**DOI:** 10.1371/journal.pntd.0006022

**Published:** 2017-11-06

**Authors:** Mary Lorraine S. Mationg, Catherine A. Gordon, Veronica L. Tallo, Remigio M. Olveda, Portia P. Alday, Mark Donald C. Reñosa, Franziska A. Bieri, Gail M. Williams, Archie C. A. Clements, Peter Steinmann, Kate Halton, Yuesheng Li, Donald P. McManus, Darren J. Gray

**Affiliations:** 1 Department of Epidemiology and Biostatistics, Research Institute for Tropical Medicine, Manila, Philippines; 2 Molecular Parasitology Laboratory, Infectious Diseases Division, QIMR Berghofer Medical Research Institute, Brisbane, Australia; 3 Research School of Population Health, The Australian National University, Canberra, Australia; 4 Discipline of Epidemiology and Biostatistics, School of Public Health, University of Queensland, Brisbane, Australia; 5 Swiss Tropical and Public Health Institute, Basel Switzerland; 6 University of Basel, Basel Switzerland; 7 School of Public Health and Social Work, Queensland University of Technology, Brisbane, Australia; 8 Hunan Institute of Parasitic Diseases, World Health Organization Collaborating Centre for Research and Control on Schistosomiasis in Lake Region, Yueyang, People’s Republic of China; Vienna, AUSTRIA

## Abstract

**Background:**

Soil-transmitted helminths (STH) are the most common parasitic infections in impoverished communities, particularly among children. Current STH control is through school-based mass drug administration (MDA), which in the Philippines is done twice annually. As expected, MDA has decreased the intensity and prevalence of STH over time. As a result, the common Kato Katz (KK) thick smear method of detecting STH is less effective because it lacks sensitivity in low intensity infections, making it difficult to measure the impact of deworming programs.

**Methodology/Principal findings:**

A cross-sectional study was carried out over a four-week period from October 27, 2014 until November 20, 2014 in Laguna province, the Philippines. Stool samples were collected from 263 schoolchildren, to determine the prevalence of STH and compare diagnostic accuracy of multiplex quantitative polymerase chain reaction (qPCR) with the KK. A large discrepancy in the prevalence between the two techniques was noted for the detection of at least one type of STH infection (33.8% by KK vs. 78.3% by qPCR), *Ascaris lumbricoides* (20.5% by KK vs. 60.8% by qPCR) and *Trichuris trichiura* (23.6% by KK vs. 38.8% by qPCR). Considering the combined results of both methods, the prevalence of at least one type of helminth infection, *A*. *lumbricoides*, and *T*. *trichiura* were 83.3%, 67.7%, and 53.6%, respectively. Sensitivity of the qPCR for detecting at least one type of STH infection, *A*. *lumbricoides*, and *T*. *trichiura* were 94.1%, 89.9%, and 72.3% respectively; whereas KK sensitivity was 40.6%, 30.3%, and 44.0%, respectively. The qPCR method also detected infections with *Ancylostoma* spp. (4.6%), *Necator americanus* (2.3%), and *Strongyloides stercoralis* (0.8%) that were missed by KK.

**Conclusion/Significance:**

qPCR may provide new and important diagnostic information to improve assessment of the effectiveness and impact of integrated control strategies particularly in areas where large-scale STH control has led to low prevalence and/or intensity of infection.

## Author summary

Worldwide, two billion people are estimated to be infected with soil-transmitted helminths (STH). These infections are primarily found in low resource settings and can result in cognitive impairment and growth stunting in children. The current control method is by chemotherapy, usually during large-scale mass drug administrations (MDA); however, this does not prevent re-infection, which can occur rapidly after treatment. The currently used diagnostics lack sensitivity in low intensity infections, resulting in underreporting of STH prevalence. In order to evaluate new control programs aimed at preventing re-infection and decreasing environmental prevalence of STH, more sensitive diagnostics are required. In this study we have shown that qPCR is far more sensitive than the traditionally used Kato-Katz (KK) microscopic technique, suggesting a role for qPCR in assessing control interventions.

## Introduction

Soil-transmitted helminth (STH) infections are the most common parasitic infections among children worldwide, especially among impoverished communities [1]. This also holds true in the Philippines, where the prevalence in school-aged children reportedly was as high as 67% in 2001 [2]. A subsequent study in 2006, which served as a baseline for the Integrated Helminth Control Program (IHCP) of the Department of Health (DOH), showed a prevalence of 54% for at least one type of STH infection and 23.1% for the prevalence of heavy-intensity infections [3].

The primary effort to control STH infections, involving mass drug administration (MDA) through school-based deworming with benzimidazole anthelminthics [1, 4–5], has increased worldwide over the past ten years. The objective of MDA is to minimize transmission and reduce morbidity, which is associated with heavy infections; however, it must be repeated at regular intervals since re-infection occurs rapidly [6–8].

In the Philippines, a nationwide semiannual school-based MDA targeting pre-elementary and Grades 1–6 pupils (aged 6–12 years old) in all public elementary schools has been implemented since 2007 by the Department of Education (DepEd) in collaboration with the DOH through its IHCP [9]. To assess the impact of the IHCP, in addition to the baseline nationwide survey of STH infections, a follow-up survey was conducted in 2009. This survey showed a significant decrease in the prevalence overall (44.7%) and of heavy-intensity STH infections (19.7%) among school-aged children (6–12 years) [10]. While the prevalence appeared to have been reduced it remained higher than the 20% target recommended by the World Health Organization (WHO) to achieve morbidity control, despite several years of MDA. In 2015, a National Deworming Day programme was established to improve access to and uptake of health interventions for all school-aged children enrolled in public elementary schools in the Philippines [11].

It is important that the prevalence and intensity of STH infection is monitored rigorously to assess the effectiveness of the control programme after repeated rounds of treatment. Hence, there is a requirement to utilize highly sensitive and specific methods for detecting infected individuals.

Several microscopy-based techniques are available and widely used for identification and quantification of STH eggs and larvae. The most widely used technique is the Kato-Katz (KK) thick smear technique, recommended by the WHO for assessing both the prevalence and intensity of infection in helminth control programmes [12]. However, as KK lacks sensitivity, particularly in areas with a high proportion of light-intensity STH infections [13, 14–16], molecular approaches are increasingly being used in monitoring and surveillance [10, 14, 17–20]. A number of recent studies have shown that the sensitivity of molecular-based diagnosis is considerably higher than the KK procedure, especially if the infection intensity is low [15, 19, 21, 22].

We conducted a parasitological survey among schoolchildren in the province of Laguna located in the Calabarzon region of Luzon in November 2014, where the reported STH prevalence in 2002 was 84.2% [23]. The aims of this study were to 1) quantify the prevalence of STH among elementary schoolchildren–particularly before the implementation of the National School Deworming Day programme on July 29, 2015; and 2) compare the diagnostic performance of KK and a multiplex quantitative real time polymerase chain reaction (qPCR) method for the diagnosis of STH infections.

## Methods

### Study design

A cross-sectional study was carried out over a four-week period from October 27, 2014 until November 20, 2014 in Laguna province, the Philippines, to determine the prevalence of STH infections among schoolchildren using the KK method and a qPCR assay.

### Ethical consideration

The study protocol was submitted to and approved by the Institutional Review Board of the Research Institute for Tropical Medicine (RITM) with approval number 2013–15 and the QIMR Berghofer Medical Research Institute (QIMRB) Human Ethics Committee (approval number: P1271). Permission was sought from the DepEd prior to the conduct of the study. With the permission obtained, we provided an orientation about the study to the principals of each school involved. Written informed consent was obtained from the parents and/or legal guardians of students invited to participate in the study. The purpose and procedures of the study were also explained to the participating children. At study completion, parasitological results were communicated to all parents, with a recommendation for treatment from the local health centre.

### Study setting and population

**Fig 1:**
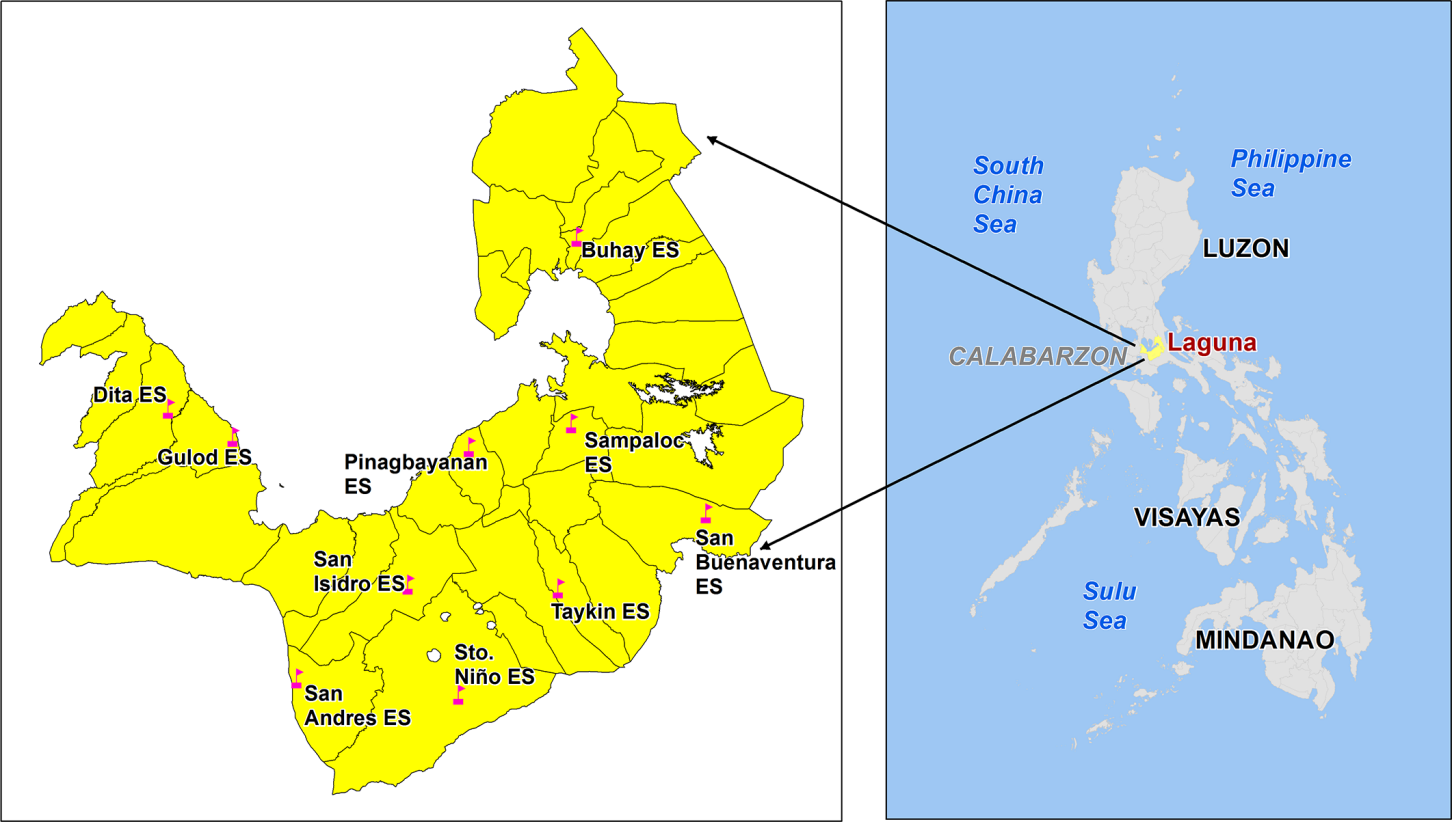
Map of the Philippines showing the location of the elementary school study sites in Laguna province. Map generated through ArcGIS 10.4 software and obtained from the National Mapping and Resource Information Authority (NAMRIA), Philippines. NOTE: the geospatial layers (i.e., Indicative Municipal Boundary by Philippine Statistical Authority) and the location of schools by Department of Education which were used to generate the map for this study was obtained from the National Mapping and Resource Information Authority (NAMRIA) through the Philippine Geoportal. This data was in a shapefile format and can be accessed through the http://www.geoportal.gov.ph.

The study was undertaken in grade 4 and 5 schoolchildren in ten selected elementary schools across ten municipalities of Laguna Province (chosen municipalities also correspond to the school districts). The selection of the municipalities was based on the rural/urban classification. This classification was based on the number of barangays classified by the Philippine Statistical Authority (PSA) as rural/urban. A municipality was classified as rural if majority of the component barangays were classified as rural. The same applies in classifying urban municipalities. Five rural (Alaminos, Calauan, Liliw, Luisiana, Siniloan) and five urban (Cabuyao, Pagsanjan, Pila, San Pablo and Sta. Rosa) municipalities were randomly selected. In this study, the school district or a cluster of school districts was considered as a homogeneous area [12] since all the selected schools are located in a dry region at low altitude. From each school district, one school was randomly selected. The selected schools were as follows: San Andres Elementary School (ES) in Alaminos, San Isidro ES in Calauan, Taykin ES in Liliw, San Buenaventura ES in Luisiana, Buhay ES in Siniloan, Gulod ES in Cabuyao, Sampaloc ES in Pagsanjan, Santo Niño ES in San Pablo, Pinagbayanan ES in Pila and Dita ES in Sta. Rosa (Fig 1).

The selection of the subjects was based on WHO recommended guidelines ([12], with some modifications). The recommended sample size was 200–250 individuals in each ecologically homogenous area to evaluate the prevalence and intensity of STH infection, with an addition of 30% to compensate for non-compliance in the submission of stool samples. A total of 325 individuals were randomly selected and invited to participate to ensure the enrolment of 250 children. A minimum of thirty-five children per school were invited. For this study, Grade 4 students (aged 9–10 years) were targeted; however, in schools where the number of Grade 4 student was less than 35, Grade 5 students (aged 10–11 years) were also included.

De-worming in the selected schools is done every January and July of the year. The parasitological survey was conducted in October-November 2014, three months after the July de-worming round. The coverage of this round is unknown, although the recorded coverage in the neighboring municipality of Calamba was only 35%. Information on the previous parasitological burden has not been examined in this area.

### Field and microscopy procedures

Stool cups were provided to the children one week before the survey week, which was allotted during five school days, and the method of collecting the samples was explained. This was to ensure submission of the stool samples on any of the five days of the survey. One stool sample per student was collected. The samples were collected, processed at the school site within two hours after collection, and read the same day using triplicate Kato-Katz (KK) thick smears (41.7 mg of stool/smear) [24]. A team of trained microscopists read the slides. The microscopists worked independently of each other on the samples assigned to them (i.e., one sample examined by one microscopist only). The slides were read at the school between 2–4 hours post collection to maximize hookworm diagnosis. The number of STH eggs was counted and recorded for each helminth species separately. To ensure validity and accuracy of the results, 42% percent of all slides were randomly selected and re-examined by a reference microscopist at the National Reference Laboratory for Parasitology at RITM.

In addition, a specimen of two to three grams of each stool sample was transferred to a 15 ml plastic tube and stored in 80% (v/v) ethanol at 4°C. The ethanol-preserved samples were transported at room temperature to QIMRB (Australia) and stored at 4°C prior to DNA isolation and subsequent molecular analysis.

### DNA extraction

DNA was extracted from the stool samples using Maxwell 16 LEV Plant DNA kits (Promega Corporation, Madison, WI USA), in conjunction with the Maxwell 16 robot (Promega). Approximately 200 mg of stool was added to a 2 mL twist cap tube and 500 μl of ROSE buffer [10 mM Tris (pH 8.0), 300 mM EDTA (pH 8.0), 1% w/v sodium dodecyl sulfate (SDS) and 1% w/v polyvinylpolypyrrolidone (PVPP)], and 1 g of 0.5 mm silica/zirconia beads (Daintree Scientific, St. Helens, Australia) was added to the tube [19, 25]. Tubes were left overnight at 4°C. The next day, tubes were placed into a Precellys tissue homogenizer (Bertin instruments, Paris, France) for 30 seconds at 6500 rpm. Following homogenization, tubes were placed in a heating block for ten minutes at 95°C, vortexed, and then centrifuged for five minutes at 10,000 *g*. Two hundred μl of H_2_0 and 300 μl of the supernatant from the centrifuged stool were added to the first well of the LEV cartridge (from the Maxwell kit). Cartridges were then placed in the Maxwell 16 robot, along with elution tubes containing 50 μl of elution buffer. The DNA extraction program for the plant kit was selected on the robot, and DNA extraction was fully automated from this point. Once completed, cartridges were discarded. DNA concentration and quality was tested using a Biotek powerwave HT Microplate Spectrophotometer. All aliquots of DNA were then diluted by a factor of five and used as the template in the resulting qPCRs. DNA with a concentration of less than 10 ng/μl were not used further.

### Multiplex PCR

Two qPCR assays were performed, a multiplex designed to identify hookworm (*Ancylostoma* spp. and *N*. *americanus*), *A*. *lumbricoides*, and *T*. *trichiura*, and a singleplex designed to identify *S*. *stercoralis*. The multiplex and singleplex qPCRs were performed utilising previously published primers and probes (Table 1). The multiplex qPCR was made up to a total volume of 25 μl that contained: 10 μl of iTaq supermix (Bio-Rad Laboratories, Hercules, California USA), 2.1 μl of H_2_O, 3 μl of DNA, 60 nM each of *A*. *lumbricoides* primers (forward and reverse) and probe (FAM), 200 nM each of *Ancylostoma* spp. primer and probe (Cy5), 200 nM each of *N*. *americanus* primers, 100 nM of *N*. *americanus* probe (ROX), 60 nM each of *T*. *trichiura* primers, and 100 nM of *T*. *trichiura* probe (Cy5.5). The qPCR was performed on a corbett rotorgene 6000 (Qiagen, Hilden, Germany). Cycling conditions consisted of two minutes at 98°C followed by 40 cycles of 98°C for 20 seconds, 74°C for 20 seconds, and 58°C for 20 seconds, followed by a final dissociation phase of 72°C for five minutes.

**Table 1:** Primers and probes used in the multiplex qPCR.

STH	Target	Reference	Name	Probe flurophore	Sequence (5' - 3')	Final concentration (nM)	From 10μM working stock conc. (μl)
*Ascaris lumbricoides*	ITS1	[20, 26]	AscF	*FAM/ZEN*	GTAATAGCAGTCGGCGGTTTCTT	60	0.096
			AscR		GCCCAACATGCCACCTATTC	60	0.096
			AscP		TTGGCGGACAATTGCATGCGAT	100	0.15
*Ancylostoma* spp.*	ITS2	[20, 26]	AncF	*Cy5/BHQ2* (LNA)	GAATGACAGCAAACTCGTTGTTG	200	0.3
			AncR		ATACTAGCCACTGCCGAAACGT	200	0.3
			AncP		ATCGTTTACCGACTTTAG	200	0.3
*Necator americanus*	ITS2	** **[20, 26]	NamF	*HEX/BHQ1* (LNA)	CTGTTTGTCGAACGGTACTTGC	200	0.3
			NamR		ATAACAGCGTGCACATGTTGC	200	0.3
			NamP		CTGTACTACGCATTGTATAC	100	0.15
*Trichuris trichiura*	ITS1	[22]	TtrF	*ROX/Iowa Black*	TCCGAACGGCGGATCA	60	0.096
			TtrR		CTCGAGTGTCACGTCGTCCTT	60	0.096
			TtrP		TTGGCTCGTAGGTCGTT	100	0.15
*Strongyloides stercoralis*	18S rRNA	[27]	StrF	*FAM/BHQ2*	GGGCCGGACACTATAAGGAT	100	0.15
			StrR		TGCCTCTGGATATTGCTCAGTTC	100	0.15
			StrP		ACACACCGGCCGTCGCTGC	100	0.15

*A. duodenale or A. ceylanicum

The singleplex qPCR was made up to a total volume of 16 μl that contained: 8 μl of GoTaq (Promega), 4.64 μl of H_2_O, 100 nM each of *S*. *stercoralis* primers and probe. The qPCR was performed on a CFX384 (Bio-Rad). Cycling conditions were the same as for the multiplex qPCR (above).

Positive and negative controls were used in each run. Two types of positive controls were used. For *Ancylostoma* spp., *N*. *americanus and A*. *lumbricoides*, cloned copies of 300 bp G-block gene fragments (purchased from Integrated DNA Technologies; IDT, Coralville, USA), specific for the gene of interest from each species were used [25]. In addition, DNA samples, extracted from eggs and adults of *Ancylostoma* spp., *N*. *americanus*, *A*. *lumbricoides*, *T*. *trichiura* and *S*. *stercoralis*, were provided by Professor James McCarthy, QIMRB. Negative controls were no-template controls, where water was used in place of DNA template.

### Statistical analysis

Statistical analyses were carried out using Stata version 13.1 and Microsoft Excel. Only schoolchildren with a matching set of three slides of KK thick smears and qPCR results were included in the final analysis. The prevalence of helminth infections, including the 95% confidence intervals (95% CIs) derived from the KK, qPCR and the combined results of both techniques, were calculated using the proportion command in Stata. The average number of helminth eggs per gram of stool (EPG) was obtained by multiplying the number of helminth eggs recorded in the KK thick smear by a factor of 24, summing the results and dividing it by the number of slides. Classification into light, moderate and heavy infection intensity was based on the average individual EPG derived from the three KK slide readings, considering thresholds set forth by WHO: *A*. *lumbricoides* [light (1–4,999 EPG), moderate (5,000–49,999 EPG), heavy (≥ 50,000 EPG)]; *T*. *trichiura* [light (1–999 EPG), moderate (1000–9,999), heavy (≥ 10,000 EPG)]; hookworm [light (1–1999 EPG), moderate (2,000–3,999 EPG), heavy (≥ 4,000 EPG)] [12, 28].

The geometric mean EPG (GMEPG) in infected persons was also calculated for each STH species. In addition, the classification of infection prevalence using the cycle threshold (Ct) values derived from the qPCR data was performed using the cut-offs as previously described [25]. The analysis using the cut-off values for intensity of infection was not calculated as this methodology is still evolving.

The diagnostic accuracy parameters including 95% CIs, were calculated using two different approaches. The first used the direct method comparison where the relative sensitivity and specificity of the KK compared to the qPCR was calculated using the qPCR as the reference standard. For the second, the relative sensitivity and specificity of both diagnostics techniques were calculated using the combined results of the KK and qPCR as reference standard. The pairwise agreement between the diagnostic techniques (KK v qPCR) was evaluated using Cohen’s kappa statistics at 95% CIs. Only species with above 20% prevalence were analyzed. The k-statistics were interpreted as <0.00, poor agreement; 0.00–0.20, slight agreement; 0.21–0.40, fair agreement; 0.41–0.60, moderate agreement; 0.61–0.80, substantial agreement; 0.81–1.00, almost perfect agreement [29]. The association of sex, age group and school with the STH prevalence derived from KK, qPCR and the combined results of both diagnostics techniques were analysed by using Chi-square test, and P values <0.05 were considered statistically significant.

## Results

### Inclusion and exclusion criteria

From the 382 schoolchildren who provided consent, 285 (74.6%) submitted stool samples. The qPCR was performed on stool samples from 264 schoolchildren due to the insufficient amount of faeces provided by the other 21. Of the 264 tested by qPCR, 263 (99.62%) had complete data records (matched triplicate KK thick smears and qPCR results). Among these 263, more than half (54%) were female and the majority (56%) were between 8–9 years old.

### Prevalence of STH stratified by diagnostic methods

We compared the prevalence of STH infection based on the results of three KK thick smears, the qPCR and the combined results of both techniques. As shown in Table 2, 33.8% of the schoolchildren studied using KK had one or more STH infection. *T*. *trichiura* was the most prevalent, infecting 23.6% of the schoolchildren; 20.5% had *A*. *lumbricoides*, and 1.9% were infected with *Enterobius vermicularis*. Applying the results from the qPCR technique, the prevalence of at least one type of STH infection was 78.3%, 60.8% for *A*. *lumbricoides*, 38.8% for *T*. *trichiura*, 6.8% for hookworm (for 4.6% *Ancylostoma* spp. and 2.3% for *N*. *americanus*), and only 0.8% for *S*. *stercoralis*. Almost three times (160/54) the number of *A*. *lumbricoides* infections and 1.6 (102/62) times the number of *T*. *trichiura* infections were determined by the qPCR technique compared with the KK method. *S*. *stercoralis*, *N*. *americanus* and *Ancylostoma* spp. were detected using the qPCR but not by KK. The number of samples that tested negative by the KK was three-fold (174/57) higher than the number testing negative by qPCR. Considering the combined results of both techniques, the prevalence of at least one type of helminth infection was 83.3%. *A*. *lumbricoides* was present in 67.7% of the schoolchildren, while 53.6% were infected with *T*. *trichiura*.

**Table 2:** Prevalence of helminth infections in 263 schoolchildren in Laguna province, Philippines, stratified by diagnostic approach.

Parasite species	Three KK slides		qPCR			Combined results of KK & qPCR	
	No.	% infected (95% CI)	No.	% infected (95% CI)	Ct cut off values for positive samples^c^	No.	% infected (95% CI)
At least one type of STH infection^a^	89	33.8 (28.34–39.81)	206	78.3 (72.90–82.92)	-	219	83.3 (78.22–87.33)
*A*. *lumbricoides*	54	20.5 (16.05–25.88)	160	60.8 (54.76–66.58)	21.28–34.73	178	67.7 (61.75–73.09)
*T*. *trichiura*	62	23.6 (18.80–29.11)	102	38.8 (33.04–44.84)	8.35–34.91	141	53.6 (47.52–59.59)
*Ancylostoma* spp.	0	-	12	4.6 (2.59–7.88)	24.74–34.68	12	4.6 (2.59–7.88)
*N*. *americanus*	0	-	6	2.3 (1.02–5.00)	25.16–32.38	6	2.3 (1.02–5.00)
*S*.*stercoralis*^b^	0	-	2	0.8 (0.19–3.08)	29.77–33.67	2	0.8 (0.19–3.08)
*E*. *vermicularis*	5	1.9 (0.78–4.50)	0	-	-	5	1.9 (0.78–4.50)

^a^At least one type of STH infection denotes any infection of *A*. *lumbricoides*, *T*. *trichiura*, the two species of Hookworm *(N*. *americanus and Ancylostoma* spp.*) and S*. *stercoralis; not including E*. *vermicularis*

^b^ qPCR to identify *S*. *stercoralis* was only performed on 257 samples with matched triplicate KK slides.

^c^ Ct values = cycle threshold scores

The prevalence of *A*. *lumbricoides* and *T*. *trichiura* by diagnostic technique was stratified according to sex, age group and school (Table 3). No significant differences were observed between sex, age group and prevalence across the three parameters for both *A*. *lumbricoides* and *T*. *trichiura*.

**Table 3:** Prevalence of *A*. *lumbricoides* and *T*. *trichiura* in 263 schoolchildren in selected elementary schools in Laguna province, Philippines as determined by the KK method and qPCR technique, stratified by sex, age and school.

	N	*Ascaris lumbricoides*						*Trichuris trichiura*					
		KK		qPCR		Both techniques		KK		qPCR		Both techniques	
		No of +tives	Prevalence (95% CI)	No of +tives	Prevalence (95% CI)	No of +tives	Prevalence (95% CI)	No of +tives	Prevalence (95% CI)	No of +tives	Prevalence (95% CI)	No of +tives	Prevalence (95% CI)
Total Examined	263	54	20.5% (16.05–25.88)	160	60.8% (54.76–66.58)	178	67.7% (61.75–73.09)	62	23.6% (18.80–29.11)	102	38.8% (33.04–44.84)	141	53.6% (47.52–59.59)
**Sex**													
Male	122	25	20.5% (14.16–28.70)	71	58.2% (49.14–66.72)	79	64.6% (55.76–72.80)	30	24.6% (17.67–33.12)	49	40.2% (31.73–49.21)	69	56.6% (47.52–65.17)
Female	141	29	20.6% (14.62–28.13)	89	63.1% (54.76–70.75)	99	70.2% (62.05–77.25)	32	22.7% (16.45–30.43)	53	37.6% (29.90–45.95)	72	51.1% (42.75–59.31)
**Age group**													
8–9 yrs	148	26	17.7% (12.19–24.64)	92	62.2% (54.00–69.68)	99	66.9% (58.82–74.07)	29	19.6% (13.91–26.87)	61	41.2% (33.48–49.40)	77	52.0 (43.90–60.04)
10–13 yrs	115	28	24.3% (17.27–33.15)	68	59.1% (49.79–67.84)	79	68.7% (59.52–76.60)	33	28.7% (21.07–37.75)	41	35.7% (27.33–44.93)	64	55.7% (46.34–64.57)
**School**													
**Rural Municipalities**													
San Andres ES	25	3	12.0% (3.59–33.27)	4	16.0% (5.69–37.54)	7	28.0% (13.20–49.84)	3	12.0% (3.59–33.27)	20	80.0% (58.25–91.97)	20	80.0% (58.25–91.97)
San Isidro ES	27	4	14.8% (5.29–35.10)	25	92.6% (72.83–98.31)	25	92.6% (72.83–98.31)	7	25.2% (12.24–46.75)	5	18.5% (7.45–39.08)	11	40.7% (23.23–60.96)
Taykin ES	15	2	13.3% (2.77–45.37)	6	40.0% (17.14–68.23)	8	53.3% (26.59–78.28)	3	20.0% (5.62–51.16)	4	26.7% (9.04–57.06)	5	33.3% (12.90–62.78)
San Buenaventura ES	36	4	11.1% (4.02–27.13)	21	58.3% (41.10–73.74)	21	58.3% (41.10–73.74)	3	8.3% (2.55–23.93)	22	61.1% (43.73–76.05)	23	63.9% (46.40–78.32)
Buhay ES	37	11	29.7% (16.80–46.98)	28	75.7% (58.59–87.24)	31	83.8% (67.37–92.81)	14	37.8% (23.26–54.99)	22	59.5% (42.42–74.48)	27	72.9% (55.77–85.25)
**Urban Municipalities**													
Gulod ES	35	7	20.0% (9.46–37.40)	20	57.1% (39.73–72.94)	22	62.9% (45.13–77.68)	14	40.0% (24.65–57.59)	13	37.1 (22.31–54.86)	22	62.9% (45.13–77.68)
Sampaloc ES	20	4	20.0% (6.99–45.36)	15	75.0% (49.74–90.09)	15	75.0% (49.74–90.09)	3	15.0% (4.39–40.37)	3	15.0% (4.39–40.37)	6	30.0% (13.06–54.99)
Sto. Niño ES	28	7	25.0% (11.81–45.34)	16	57.1% (37.51–74.75)	17	60.7% (40.77–77.62)	4	14.3% (5.11–33.99)	6	21.4% (9.43–41.65)	9	32.1% (16.90–52.45)
Pinagbayanan ES	20	10	50.0% (27.68–72.31)	12	60.0% (36.01–79.98)	18	90.0% (64.48–97.80)	10	50.0% (27.68–72.31)	4	20.0% (6.99–45.36)	14	70.0% (45.00–86.93)
Dita ES	20	2	10.0% (2.19–35.51)	13	65.0% (40.42–83.55)	14	70.0% (45.00–86.93)	1	5.0% (0.57–32.27)	3	15.0% (4.39–40.37)	4	20.0% (6.99–45.36)

CI, confidence Interval.

School prevalence for *A*. *lumbricoides* ranged from 10% (95% CI: 2.19–35.51) to 50% (95% CI: 27.68–72.31) as determined by the KK; 16% (95% CI: 5.69–37.54) to 92.59% (95% CI: 72.83–98.31) by qPCR; and 28% (95% CI: 13.21–49.85) to 92.59% (95% CI: 72.83–98.31) by the combined results of both techniques. For *T*. *trichiura*, the school prevalence ranged from 5% (95% CI: 0.57–32.27) to 50% (95% CI: 27.68–72.31) as detected by KK; 15% (95% CI: 4.39–40.37) to 80% (95% CI: 58.25–91.97) by qPCR and 20% (95% CI: 6.99–45.36) to 80% (95% CI: 58.25–91.97) by both techniques. Pinagbayanan ES had the highest prevalence of both *A*. *lumbricoides* (50%; 95% CI: 27.68–72.31) and *T*. *trichiura* (50%; 95% CI: 27.68–72.31) following the results of the KK. Meanwhile, San Isidro ES had the highest prevalence of *A*. *lumbricoides* infection by qPCR (92.6%; 95% CI: 72.83–98.31) while San Andres ES had the highest prevalence of *T*. *trichiura* infection (80%; 95% CI: 58.25–91.97), also determined by qPCR. The differences observed among schools for the prevalence of *A*. *lumbricoides* (KK *P* = 0.031; qPCR *P* = <0.001, combined results of both techniques *P* = <0.001) and *T*. *trichiura* (KK *P* = <0.001; qPCR *P* = <0.001, combined results of both techniques *P* = <0.001) were statistically significant across the three parameters.

### Intensity of infection characteristics

The geometric mean EPG values for *A*. *lumbricoides* and *T*. *trichiura* obtained by the KK technique and the mean Ct values derived from the qPCR are summarized in Table 4, with the EPG count range (KK), the Ct range found in a single stool sample (qPCR), and the infection intensities among the positives (KK) stratified by infection intensity category defined by WHO.

**Table 4:** Characteristics of STH infections among schoolchildren in selected elementary schools in Laguna province, Philippines, as determined by the KK method and qPCR technique.

Parasite species	No. of children examined (with matched 3 KK slides and qPCR)	Kato-Katz							qPCR	
		No. (%) of children infected	No. (%) of children infected with egg count readings	Range of EPG counts^a^	No. of infected children stratified by infection intensity (values in brackets are percentages, %)			Geometric mean EPG (range)	No. (%) of children infected	Mean Ct values (range)^b^
					Light	Moderate	Heavy			
*A*. *lumbricoides*	263	54 (20.53%)	52 (19.77%)	8–106,560	25 (48.1)	23 (44.2)	4 (7.7)	8.26 (2.07–11.57)	160	29.48 (21.28–34.73)
*T*. *trichiura*	263	62 (23.57%)	61 (23.19%)	8–3,976	52 (85.2)	9 (14.8)	0	5.40 (2.07–8.28)	102	17.34 (8.35–34.91)

^a^ EPG = eggs per gram of feces based on KK thick smear examination

^b^ Ct values = cycle threshold scores, cut-offs for the intensity of infection was available only for *A*. *lumbricoides*. Can’t calculate Ct intensity for *T*. *trichiura*

According to the WHO-defined infection intensity classification [12, 28], the majority of infected schoolchildren included in the final analyses had low intensity infections: 48.1% (25/52) for *A*. *lumbricoides*, and 85.2% (52/61) for *T*. *trichiura* (Table 4). Based on the Ct values derived from the qPCR analysis, the mean Ct values were 29.48 (range: 21.28–34.73) for *A*. *lumbricoides* and 17.34 (range: 8.35–34.91) for *T*. *trichiura* (Table 4).

### Relative sensitivity and specificity; and agreement of the two diagnostic methods

A comparison of diagnostic accuracy was only performed for *A*. *lumbricoides* and *T*. *trichiura* because of the low number of individuals harbouring the other helminths.

The relative sensitivity of the KK for detecting at least one type of STH infection using the qPCR as the reference standard was 36.9% (95% CI: 30.3% - 43.9%). Relative specificity was calculated as 77.2% (95% CI: 64.2% - 87.3%). Further, the relative sensitivity of the KK for detecting *A*. *lumbricoides* was calculated as 22.5% (95% CI: 16.3% - 29.8%) and 22.3% (95% CI: 14.7% - 31.6%) for *T*. *trichiura*. The calculated relative specificity was 82.5% (95% CI: 73.8%-89.3%) for *A*. *lumbricoides* and 75.8% (95% CI: 68.4–82.2%) for *T*. *trichiura*.

Following the combined results of both techniques (triplicate KK thick smears and the qPCR) as reference standard (Table 5), the relative sensitivity for detecting at least one type of helminth infection was higher using qPCR than the KK (94.1% by qPCR versus 40.6% by KK). The relative sensitivity for *A*. *lumbricoides* diagnosis was higher for qPCR than KK (89.9% by qPCR versus 30.3% by KK). For the diagnosis of *T*. *trichiura*, similarly, PCR showed a better relative sensitivity than KK (72.3% by qPCR versus 44% by KK). The calculated relative specificity for both techniques for all species was 100%.

**Table 5:** Diagnostic accuracy of triplicate thick smear KK and qPCR using the combined results of the KK and qPCR as reference standard in stool samples from 263 schoolchildren in Laguna province, Philippines.

Parameter	Test	STH species	Sensitivity, % (95% CI)
Combination of methods (results of KK and qPCR) as reference standard	KK	At least one type of STH infection	40.6% (34.1% - 47.5%)
	qPCR		94.1% (90.1% - 96.8%)
	KK	*A*. *lumbricoides*	30.3% (23.7% - 37.7%
	qPCR		89.9% (84.5% - 93.9%)
	KK	*T*. *trichiura*	44.0% (35.6% - 52.6%
	qPCR		72.3% (64.2% - 79.5%)

Parasite prevalence agreement statistics (Table 6) showed a slight agreement (κ = 0.0425) between triplicate KK thick smears and the qPCR for *A*. *lumbricoides* egg detection while a poor agreement was found for *T*. *trichiura* (κ = - 0.0180). The agreement between the two techniques for each helminth species, however, was not significant (*A*. *lumbricoides P* = 0.1624; *T*. *trichiura P* = 0.6224). For all the parasites analyzed, the qPCR detected a large number of samples not detected by KK (*A*. *lumbricoides*, 124; and *T*. *trichiura*, 79). However, a small percentage of samples were observed to be positive by KK for *A*. *lumbricoides* 7% (18/263) and *T*. *trichiura* 15% (39/263) but negative by qPCR.

**Table 6:** Parasite prevalence agreement statistics between triplicate thick smear KK and qPCR for the diagnosis of STH infections in stool samples from 263 schoolchildren in Laguna province, Philippines.

STH infection	qPCR	Triplicate KK smears		Total Agreement (%)	Kappa	SE of Kappa
		Pos	Neg			
*A*. *lumbricoides*	Pos	36	124	121 (46.01)	0.0425	0.0431
	Neg	18	85			
*T*. *trichiura*	Pos	23	79	145 (55.13)	- 0.0180	0.0579
	Neg	39	122			

### Co-infections

**Fig 2:**
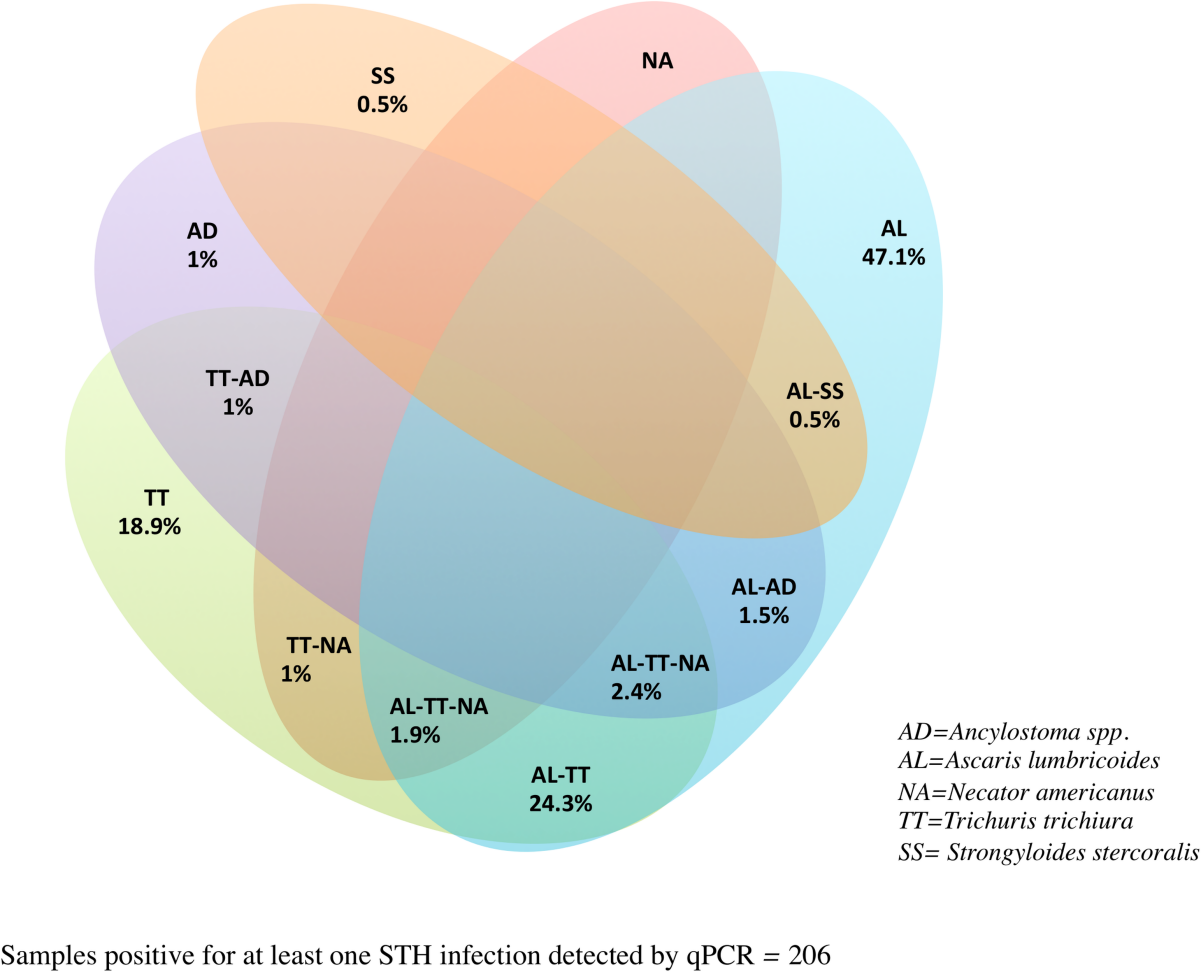
Venn diagram detailing the specific division of STH co-infections determined by qPCR.

In terms of detecting samples with co-infections, there was a two-fold (67/27) increase in the number of samples with two or more parasite species detected by qPCR than by KK. Of the 206 schoolchildren positive for at least one type of STH infection detected using the qPCR, 28.2% had two different parasites while only 4.4% harboured three different worm species (Table 7). The various helminth parasite combinations are shown in Fig 2. Co-infections with *A*. *lumbricoides* and *T*. *trichiura* were the most prevalent (24.3%) co-infection observed in this study.

**Table 7:** Distribution of single, double and triple STH infections among schoolchildren from selected elementary schools in Laguna province, Philippines positive for at least one STH infection by KK and qPCR.

Number of parasite infections	KK (total positives = 89)		qPCR (total positives = 206)	
	No	% infected (95% CI)	No	% infected (95% CI)
Single	62	69.7% (59.15–78.45)	139	67.5% (60.72–73.56)
Double	27	30.3% (21.54–40.84)	58	28.1% (22.39–34.73)
Triple	0	-	9	4.4% (2.27–8.22)

### Quality control of microscopy reading

Slide reading validation showed an overall sensitivity of 97.6% (95% CI: 91.5% - 99.7% and specificity of 85.7% (95% CI: 67.3% - 96%) for detecting STH. Sensitivity for *A*. *lumbricoides* was 96% (95% CI: 86.3% - 99.5%), whereas the sensitivity for *T*. *trichiura* was 98.2% (95% CI: 90.6% - 100%).

## Discussion

This study assessed the prevalence of STH infections among schoolchildren in the Philippine province of Laguna, and showed that a qPCR method was far more sensitive than copro-microscopy using triplicate KK thick smears. Indeed, there was a marked difference in prevalence obtained between the two techniques for at least one type of STH infection (33.8% by KK vs. 78.3% by qPCR), *A*. *lumbricoides* (20.5% by KK vs. 60.8% by qPCR) or *T*. *trichiura* (23.6% by KK vs. 38.8% by qPCR). Other helminth species found by the qPCR method that were missed by KK were hookworms (*N*. *americanus* and *Ancylostoma* spp.; 6.8%) and *S*. *stercoralis* (0.8%). Combining the results obtained with the qPCR and the triplicate KK thick smears, the observed prevalence for at least one type of helminth infection, *A*. *lumbricoides* or *T*. *trichiura*, was 83.3%, 67.7% and 53.6%, respectively.

We used two approaches to determine the diagnostic accuracy of the KK and qPCR: (1) a direct method assessment for determining the relative sensitivity and specificity of the KK and (2) use of the combined results of the KK and qPCR procedures. The relative diagnostic sensitivity of the KK using the two approaches was shown to be considerably lower in detecting at least one STH infection, *A*. *lumbricoides* or *T*. *trichiura*. The current study shows also that the qPCR outperforms the KK for STH diagnosis, a feature very much in line with the findings of previous studies [15, 19, 21, 22]. The agreement statistics also replicate our finding regarding the relative sensitivity of the two techniques with the KK showing poor agreement with the qPCR for *T*. *trichiura* (kappa: -0.0180; *P* = 0.1624) and slight agreement for *A*. *lumbricoides* (kappa: 0.0432; *P* = 0.1624).

Although the qPCR had better relative diagnostic sensitivity compared with the triplicate KK thick smears, it failed to detect some infections, as a number of individuals were found positive by the KK but negative in the qPCR. However, these false-negatively diagnosed individuals with *A*. *lumbricoides* and *T*. *trichiura* in the qPCR did not have lower EPG values in the KK, suggesting that there must have been an additional factor that affected the sensitivity of the DNA-based method. For 86 stool samples, there was less than 200 mg available for DNA extraction and subsequent qPCR, which might be one possible explanation for a loss of sensitivity in some samples.

The KK did not detect any hookworm species, while the qPCR showed a prevalence of 4.6% of *Ancylostoma* spp. and 2.3% of *N*. *americanus* (Table 2). Hookworm eggs lyse quickly after defecation, therefore if KK slides are not prepared and read rapidly, it is unlikely that any hookworm eggs will remain to count [30, 31].

The results from this study also showed that the qPCR was able to detect multiple helminth infections in 25% of the study population, a feature common in the Philippines [3, 13]. This may have implications for the current treatment guidelines for STH control as individuals with multiple parasitic infections tend to have a higher level of morbidity [32].

The present study shows that the prevalence of STH infections is likely to be underestimated by the diagnostic procedure, based on the KK, currently used in the Philippines. The qPCR method holds promise for more accurate helminth diagnosis than the KK technique, especially in areas with low-intensity infections. qPCR also has the potential for future use in the monitoring of STH transmission; surveillance of helminth control programmes–providing accurate information on the scaling up/down of control; and verification of local elimination. Resistance to use qPCR methods may centre around the higher cost of this technique relative to KK; however, recent work highlights that, in the context of elimination (as opposed to control) of STH, these higher up front costs are likely to be outweighed by potential longer term benefits of elimination [33]. With the scaling up and intensification of control interventions against neglected tropical diseases, leading towards elimination, it is anticipated that the prevalence and intensity of STH infections will decline in accordance with the WHO objectives set for the year 2020 [34]. However, because of this decline, improved diagnostic procedures will be required to assess whether transmission has ceased.

It is evident that the qPCR approach is superior to the KK procedure for detecting STH infections. The current guidelines for STH control may require reconsideration, especially if the KK method continues to be used as the key-monitoring tool for treatment effectiveness. The advantages of the KK–low cost and easy to perform–suggest, however, that it will continue to be the diagnostic procedure of choice for control programs in the near future.

A major aspect of the intervention process is determining the correct baseline prevalence and intensity of STH in target communities to accurately assess the impact of repeated treatments. Although qPCR cannot be performed in the field, requires costly reagent and equipment and trained technicians to undertake the test and analyze the results, it may however, provide new and important diagnostic information to improve the assessment of the effectiveness and impact of integrated helminth control strategies.

### Conclusion

The STH prevalence among schoolchildren in Laguna province obtained from this study using the combined results of the KK and qPCR procedures was 84.2%; this is similar (although using more sensitive diagnostics) to that reported in 2002 in Laguna (KK only) when the nationwide school-based MDA was not yet locally implemented [23]; and despite several years of MDA. STH control strategies in the Philippines thus need to be enhanced, including sustaining the national deworming day programme with high coverage levels and regular monitoring of its impact with the use of a more sensitive and specific diagnostic technique. The higher sensitivity of the qPCR as shown in this study would imply that the true STH prevalence is substantially higher in the Philippines and elsewhere than currently assessed. This could have a direct impact on policies and control programmes based on suggested WHO treatment guidelines. Higher prevalence estimates obtained with the qPCR method would, for example, result in an increased number of communities subjectively defined as community category II (classified as with greater than 50% prevalence of infection and low infection intensities), for which community-based MDA is recommended [12]. Furthermore, additional interventions (e.g. water, sanitation and hygiene (WASH) and a proven health education intervention (e.g. “Magic Glasses”)) [35] as part of a multi-component integrated approach will be required to augment MDA for sustainable control and even elimination of STH infections.

## Supporting information

S1 Checklist STROBE checklist. (DOC)
